# A systematic review of participatory research involving forensic mental health patients

**DOI:** 10.1186/s12888-026-07813-8

**Published:** 2026-01-23

**Authors:** Fenia Ferra, Peggy Walde, Marcel Daum, Stefan Teipel, Eva Drewelow, Olga Biernetzky, Birgit Völlm

**Affiliations:** 1 Clinic of Forensic Psychiatry, Rostock University Medical Center, Rostock, Germany; 2 Department for Psychosomatic and Psychotherapeutical Medicine, Rostock University Medical Center, Rostock, Germany; 3 Deutsches Zentrum für Neurodegenerative Erkrankungen (DZNE), Rostock/Greifswald, Germany

**Keywords:** Participatory research, Involvement, Patient participation, Forensic mental health, Secure hospitals

## Abstract

There is currently limited information and guidance on utilising participatory research within forensic mental health settings. This systematic review aimed to explore the use of participatory research within those settings, identify helpful practices, barriers and challenges, as well as explore the impact reported of participatory research in research and knowledge production. Fourteen studies and eleven other types of publications have been identified from electronic databases, PsycINFO, PubMed, Embase, Web Of Science Core Collection, Google Scholar and ProQuest and hand searches of academic journals. Thematic analysis was used for synthesising data collected. Four themes were identified: (1) Building equal relationships, (2) Speaking the same language, (3) An involving system, (4) Impact for all. Analysis showed that the use of participatory research in such settings relies on human interaction and equal co-production within commonly restrictive and coercive environments. The complexity of participatory research is also reflected in its reporting. A standardised (but also flexible) model of reporting would be valuable for future research. In addition to summarizing the findings of research in this area, we have compiled a list of *‘active ingredients’* for the successful use of participatory research in forensic mental health settings.

**Supplementary Information:**

The online version contains supplementary material available at 10.1186/s12888-026-07813-8.

## Background

 Participatory research relies on the active collaboration between researchers and community members (e.g. People With Lived Experience of certain phenomena). It allows the involvement of different members of the community (some often overheard) in research, frequently in an attempt to better inform and lead research and policy [1, 2]. It can be better understood as a research philosophy rather than simply methodology, as frequently explained in the literature (e.g [3]). Through a participatory research lens, research is a community rather than a purely academic task, which can produce knowledge, but also empower (e.g. [1–3]). The origins of participatory research can be traced back to Kurt Lewin’s [4] and Paulo Freire’s [5] work on social change and critical consciousness. Stressing the limitations of more traditional research methods, social movements gave rise to participatory research methods in researching marginalised and oppressed groups. Some early examples of participatory research are Hall’s work in Tanzania [6] and Tandon’s research in India [7], both illustrating the benefits of including ‘overheard’ voices in research and policy making and moving away from the overreliance on dominant voices.

Different approaches and terms for participatory research have been developed throughout the years: Participatory Research (PR) [8], Participatory Action Research (PAR) [9], Community-Based Participatory Research (CBPR) [10], and Patient and Public Involvement (PPI) [11]. Different terms (and approaches) have been dominant in different countries, with CBPR most frequently used by authors from the United States, PPI mostly found in United Kingdom and PR used by authors from Canada [12]. Even though there might be some differences in the theoretical foundation and aims (i.e. empowerment, action), participatory research has commonly been employed to shed light on (often) overlooked topics, broaden our understanding of phenomena, and empower community members. It has been used in different fields and has frequently involved marginalised and/or oppressed groups, such as those with severe mental health issues [1] or substance abuse [13, 14], youth with mental health issues [15], refugees and asylum seekers [16], and those in prisons [17–19], and forensic mental health settings [20–22].

Several scholars have looked into the use of participatory research in the field of mental health and have tried to identify good practices (ethical and effective strategies employed) (23, offering a historic review of participatory research in psychiatry). The notable differential in the level and type of involvement of community members in mental health research has frequently been discussed. A good example is a scoping review conducted by Levac et al. [23] exploring how participatory research has been utilised in the field of psychology. The review showed that in forensic research, community members were in most cases involved (only) in data collection and analysis rather than research design, recruitment of participants or dissemination. Similarly, Brierley-Jones et al. [24] conducted a systematic review looking at the design, development, and implementation of patient safety interventions in mental health care, including forensic mental health. The review also demonstrated that the level of involvement of community members on research varied, with less than a third of studies involving community members as partners, a third as advisors, and almost a third as co-thinkers, while only a small number (7%) of the studies involved community members as decision-makers.

Hoekstra et al. [12] conducted a review of reviews focusing on research principles and strategies used in participatory research approaches, as well as their impact. They identified overarching principles and strategies for different research stages, as well as the wider research process. Additionally, they developed some initial guidance for making use of the review’s findings on research partnerships, such as building and maintaining relationships, and selecting, and adapting principles and strategies. They noted that principles, strategies and the level and type of involvement is context specific.

While there has been a rise in the use of participatory research approaches in the field of mental health, and good practices have been identified, such as creating (equal) partnerships, paying attention to language and values systems, and being open, curious and critical to personal assumptions (e.g. by Bates [25]), limited information on what is most helpful in forensic mental health research exists. Forensic psychiatry, unlike other areas in the field of psychiatry, is not based on the traditional two-way clinician-patient interaction, but rather on a three-way relationship clinician-patient-society (managing risk) [26]. Care and therapy in forensic psychiatry are commonly imposed on individuals, leaving little room for informed decision making and participation on the patients’ side [27]. According to Völlm et al. [28], who conducted a rapid review on how to engage forensic mental health patients^1^ in research, more research in this field is imperative, as the intense power imbalances and restrictive nature of forensic contexts [30] might undermine or come into conflict with participatory research principles and practice (e.g. creating an equal partnership within a restrictive environment, where individuals are placed sometimes against their own will). At the same time, forensic mental health patients might raise unique challenges on being effectively involved in research due to severe mental health issues (sometimes affecting social interaction) and other special needs (including learning difficulties, autistic traits). There has been a big variation, and discrepancies of research methodologies and tools employed, while there have been some ethical and practical challenges identified, which are key for co-production within such restrictive settings (e.g [28]).

In summary, there is a great variation in choices and strategies employed, with community members being involved in different ways (e.g. the aim of involvement), different level of involvement^2^ (e.g. different levels or involvement or roles, i.e. advisor vs. decision-maker) and at different time points (e.g. involved in different stages). As such, it is still unclear which practices/strategies of participatory research might be helpful particularly with patients in forensic mental health hospitals. Identifying meaningful approaches to involvement is of great importance as limited involvement could lead to real or perceived tokenism^3^ [34]. Tokenism can act as a barrier to effective participatory research, as it restricts it from meeting its ultimate purpose, social change, and empowerment [35]. Sometimes marginalised groups might be able to be ‘seen’, but not effectively and fully heard, and thus equally included in the co-constructive research process, due to complex power dynamics, relations, and systemic and multiple oppression [36].

This review addresses the following questions: What is considered to be helpful with regard to participatory research in forensic mental health settings and what is the impact reported of using participatory research in such settings on PWLE, practitioners, settings, researchers and knowledge production. Therefore, we seek to examine patterns in existing research to date. Our review aims to achieve:

- An exploration of what is considered helpful in participatory research in the field of forensic psychiatry, and more particularly secure hospitals (in-patients and out-patients).
- An examination of the impact reported of participatory research in research and knowledge production in the field of forensic psychiatry.
- A better understanding of how forensic mental health patients (and/or People With Lived Experience (PWLE)) in the field of forensic psychiatry may be involved in participatory research (e.g. level and type of involvement).
- An exploration of the potential limitations and barriers of the use of participatory research in the field of forensic psychiatry (for researchers, patients, stakeholders, knowledge production, policy making and interventions).

This systematic review is part of a wider project, ‘PART-Beirat’ [37], looking at the use of participatory research methods in forensic mental health (with a particular focus on Germany) and aiming on developing guidelines for best practice.

## Methods

This systematic review (as defined by [38]) adheres to the Preferred Reporting Items for Systematic Reviews and Meta-Analyses (PRISMA) statement [39], and it was registered with PROSPERO (CRD42022363750).

### Context

The context of focus was secure hospitals or psychiatric units within the prison system^4^.

### Phenomena of interest

This review sought to explore participatory research approaches, helpful strategies that can be applied in this context and the impact reported thereof on PWLE, practitioners, researchers, settings and knowledge production.

### Information sources

We conducted a search of peer-reviewed articles, dissertations, reports and grey literature using PsycINFO, PubMed, Embase, Web Of Science Core Collection, Google Scholar and ProQuest in October 2022. Using our main keywords, we have checked the first five pages of Google Scholar and ProQuest. Additionally, hand searches were conducted on the reference lists of identified literature and leading journals in the field of participatory research and forensic psychiatry: Action Research, Research Involvement and Engagement, International Journal of Forensic Mental Health and Journal of Forensic Psychiatry and Psychology.

### Search strategy

The search strategy was developed based on relevant literature (e.g [40]), and previous work of members of the research team [28]. Terms were to capture different approaches of participatory research used in the field of forensic mental health (Table 1).

**Table 1:** Search terms

community-based participatory research (MeSh), participatory research, action research, patient oriented research, patient and public involvement/PPI, peer-led /user-led research, peer support, patients/family/stakeholders involvement
**AND**
forensic psychiatry field: forensic psych* (MeSh), secure hospital, forensic mental health, mentally ill offender*, mentally disordered offender/MDO, hospital order, prison /jail /gaol /remand/penitentiary/correctional, incarcerat*/inmate/imprisoned/detention/custody, offender/delinquent/felon/convicted, crim*, NCRMD, NGRI, Judicia*/court

### Inclusion/ exclusion criteria

Studies using participatory action research methods involving adult forensic mental health patients and professionals, conducted within secure hospitals, or working with people who have experience of being hospitalised within a secure hospital environment, both in-patients and out-patients, were included in the sample. Studies not providing information on the involvement of patients were excluded. Moreover, even though Delphi studies might constitute a type of participatory research, they were excluded from this review, due to their often less direct engagement of PWLE. Studies conducted within prison settings were excluded. However, studies involving populations from special psychiatric units/wards within prison settings were included. There were no restrictions with regards to diagnoses or severeness of the illness, interventions, intended outcomes or dates, geographical location or language used. There were also no restrictions on methodological approaches. Qualitative and mixed studies were included in the review. Grey literature, such as conference proceedings, reports, dissertations/theses and commentaries were included.

### Procedure

#### Study selection

After removal of duplicates (*n* = 1,364), study selection was conducted in two stages: title and abstract screening (Stage 1), followed by full-text screening (Stage 2). Zotero reference management software was used throughout the review process.

In Stage 1, title and abstract screening (*n* = 2,578) was primarily conducted by the first reviewer (FF) in accordance with predefined inclusion and exclusion criteria. A second reviewer (MD) independently screened a random 20% sample of records to assess consistency of study selection. Discrepancies were resolved through discussion.

**Fig. 1:**
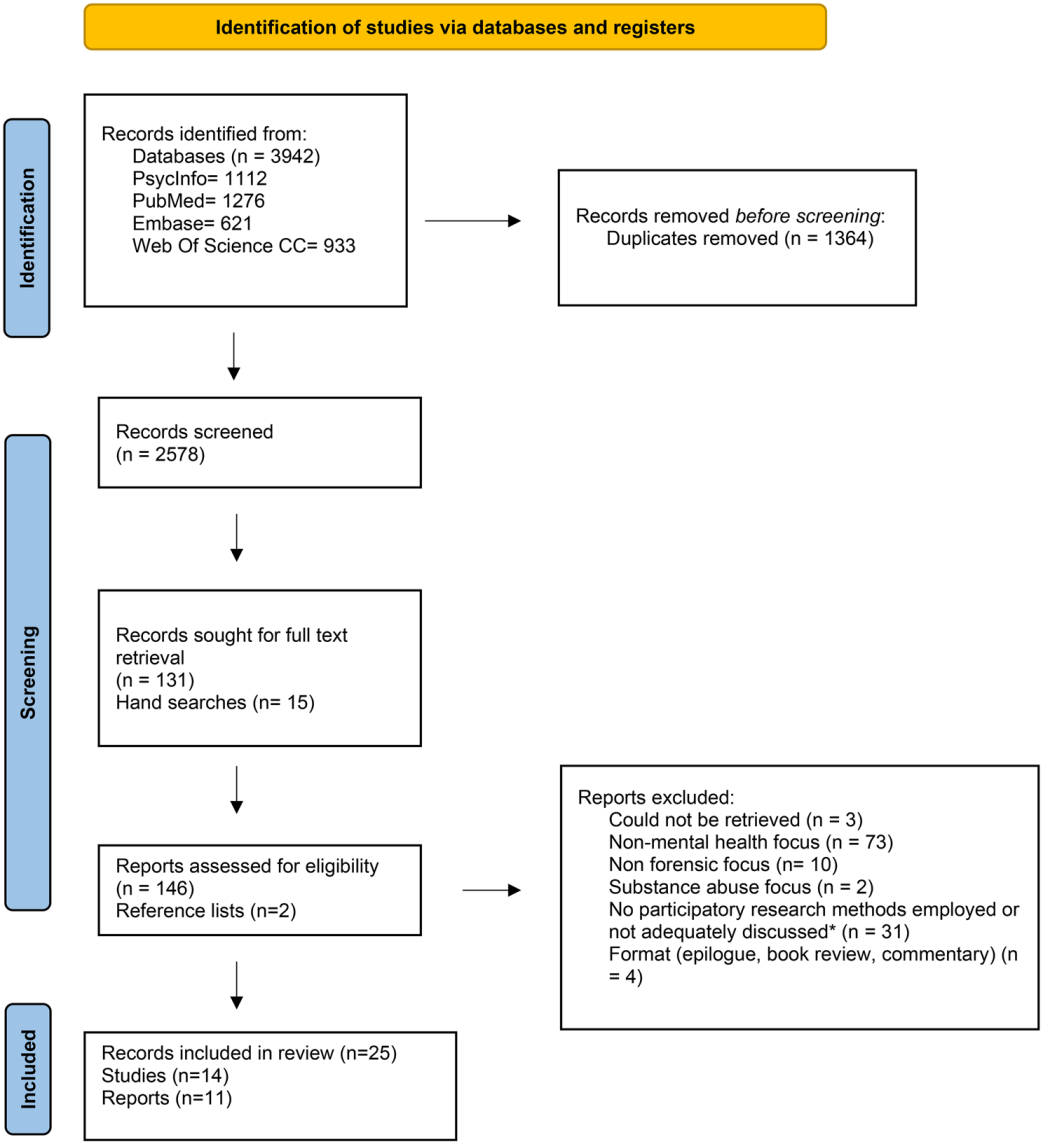
Prisma flowchart

In Stage 2, full texts of potentially eligible studies (*n* = 131), as well as studies identified through hand searching (*n* = 15), were assessed by the first reviewer (FF). The second reviewer (MD) independently screened a random 20% sample of full texts^5^, following the same procedure as in Stage 1. Studies identified through reference list screening (*n* = 2) were independently assessed by both reviewers. Disagreements were discussed and resolved by consensus. As agreement was achieved in all cases, no third reviewer was required. The final list of included studies was reviewed by the second reviewer (Fig. 1).

### Data extraction

A standardised form, piloted by FF/MD/PW/OB, was used to extract data from all studies included. Extracted data included author(s) name(s), year of publication, location of the study, setting, population, recruitment methods, methodological approach applied, length of the study, level of patient/family/stakeholder involvement, the different stage(s) they were involved in, ethical aspects/issues arising, main findings, limitations, and directions for future research. Excel was utilised for data extraction. Data were extracted by one reviewer (FF) and 20% of data were checked by a second reviewer (PW). Disagreements were discussed and resolved by consensus.

### Critical appraisal

We used two different tools to assess the quality of included studies in this review, the Critical Appraisal Skills Programme (CASP) [42] for qualitative studies and the Mixed Methods Appraisal (MMAT) [43] for one mixed methods study. Two reviewers were involved in the process, assessing all included studies. The CASP [42] includes 10 items, divided into three sections (Section “Background”. Are the results valid, Section “Methods”. What are the results, Section “Results”. Will the results help locally). The MMAT [43] includes 7 items, 2 screening questions and 5 questions specific to the study’s methodology (in this case mixed methods). Two reviewers independently reviewed the studies. Any differences were discussed and resolved.

One item that yielded most differences was item 6. Item 6 asks if the relationship between researcher and participants was adequately considered. Due to the nature of participatory research, it was sometimes difficult to distinguish between researchers and participants, while even in cases where this was clear, it was difficult to answer the question as, to some extent, participatory research always involves such considerations (such as relationships between researchers/participants, power dynamics). We decided to rate studies only based on the information they provided on the manuscripts.

All qualitative studies, except one, were found to be of moderate to high quality, similar to the mixed methods study (5***). One study [44] was found to be of moderate to low quality because of missing information (Appendix A). Other studies also lacked information on various aspects/parts of the study/methodology.

### Level of involvement

The Involvement Matrix [32] was used to explore the level of involvement in the studies included in our sample. The origin of the Involvement Matrix lies in Arnstein’s ladder of participation [31], but unlike the original participation ladder, in the Involvement Matrix there is no references to ‘levels’ of participation (i.e. a vertical, hierarchical approach) but rather ‘roles’ (i.e. a horizontal, equal approach). The tool allows to explore the different roles that community members take throughout the different stages of the research process (e.g. advisor, decision maker).

### Data synthesis

Reflexive thematic analysis was used for the analysis of data [45], following a six-step process (1) data familiarisation (2), codes generation (3) constructing descriptive themes (4), reviewing themes (5), naming and revising themes/ constructing analytical themes, and (6) writing up. Thematic analysis is a common approach for synthesising qualitative data, which has been suggested to allow researchers to synthesise data in a transparent way, whilst facilitating the development of new concepts, interpretations and the formulation of conclusions [46]. NVIVO 14 was utilised for the analysis. A second reviewer (PW) analysed 30% of the sample and later reviewed all final themes and subthemes. One of the main purposes of including a second reviewer was to enrich the analysis, and as such, any points raised by the second reviewer were discussed and later reflected in the final analysis, themes and subthemes. To achieve that, reviewers met at different points of time. Reviewers initially separately coded two studies and then met to discuss their codes (step 2, code generation). They reflected on any differences on their codes and replaced some of their codes with new ones emerging from their conversation. Similarly, reviewers coded separately 30% of the sample and met again to discuss their initial/descriptive themes and subthemes (step 3, constructing themes). During the conversation some of the themes were reshaped, and some new themes emerged. After the first reviewer developed the final themes, reviewers met again to discuss. At this stage, the second reviewer led a critical discussion on the themes, which aimed to promote reflection (step 4, constructing themes). Some of the themes and sub-themes were revised during the meeting, whilst some others were later revised by the first reviewer (step 5, naming and revising themes). Writing up of the analysis was led by the first reviewer. Reviewers met at the end and discussed the analysis, whilst proceeding in changes they thought could improve structure and meanings. All authors had the opportunity to provide reviewers with further comments and thoughts.

### Active ingredients

A *list of best practices or ‘active ingredients’*^6^, as we will be referring to as throughout this manuscript, was compiled and included here. The idea of compiling such a list was born from the need to summarise practical tips and advice to inform the later stages of our research project, as well as other research, and was further inspired by Abram et al. [47]. The ‘active ingredients’ concerned all different stages of research process, from applying for funding, submitting an ethics application, recruiting forensic mental health patients and forming a research group to training provision, research meetings, data collection and analysis. The list consists of 16 sections (overall tips, advice with regard to funding applications, ethical considerations, informed consent, PWLE’s payment and status [ie employees], advice on forming the research group, rapport building, meetings, different ways of involvement, disclosure of personal information [and challenges], mental health, training, continuous support and reflexive practice, data collection, analysis and dissemination) covering different stages and aspects of the research process. A full version of those active ingredients is available in appendices (Appendix B). The ‘active ingredients’ list was discussed and compiled by two reviewers (FF, PW). This is not part of our main data synthesis and as such, should be treated as supplementary material. The list has been included in the manuscript (Appendix B) to enhance transparency and to contribute to the broader sharing of information and knowledge.

## Results

### Sample

Fourteen studies (*n* = 14) and eleven (*n* = 11) other types of publications (i.e. reports, reviews, an editorial, secondary analysis & reflections) were included in this review. The other types of publications included one rapid review, one case study, two book chapters, one editorial, and six reports/secondary analyses/retrospective considerations or reflections on studies. There were no quantitative studies identified. There were two occasions that a study and a report refereed to the same study ([20, 48] and [49, 50]) making the number of studies included in our sample twelve (*n* = 12). From these twelve studies, eight were conducted in the UK, two in Canada, one in the Netherlands and one in New Zealand. The length of the studies ranged from 7 months to 5 years, even though several studies did not specify the length of projects. The number of forensic mental health patients involved in projects ranged from one to eight. Two studies (*n* = 2) [8, 47] involved women, all other included only male forensic patients in their research teams. None of the studies involved family members or carers. Experts/practitioners (people with a practical role in patient care) were only involved in eight studies (*n* = 8), information was lacking in two studies, while no involvement of experts or professional was included in four studies (*n* = 4). Only six studies provided information on the patients’ recruitment process, and three provided information on forensic mental health patients’ payment. Seven studies (*n* = 7) gave information on the training(s) provided as part of their projects. An overview can be found in Table 2. Table 3 consists of all other publications, reports, reviews, and reflections included in the review.

**Table 2:** Overview of studies included in the review

	Author, date	Country	Setting	Length	Involvement approach	Research team (*N*)			Recruitment strategy (PWLE)	Payment	Training (PAR)
						Researchers	PWLE	Experts			
1	Abram et al. 2019	UK	LS & community	7 months (22 sessions)	PAR	NS	5 current and 2 ex-service users	NS	convenience sampling	vouchers	Yes
2	Alred 2018	UK	Community services	5 years	Collaborative Enquiry	1	2 ex-service users	-	advertisement	Yes	Yes
3	Banongo et al. 2006	UK	University	9 months	Participatory research/user involvement in research	7	7	-	advertisement & support from HR	causal employees	Yes
4	Cook & Inglis 2012*	UK	LS & MS (disability services)	2 y, 3 months	FCAR	3 (1 withdrawn)	7 (2 withdrawn)	10	NS	N/A	Yes
5	Cook & Inglis 2008*	UK	LS & MS (disability services)	NS	FCAR	3 (1 withdrawn)	7 (2 withdrawn)	10	purposive sampling	N/A	Yes
6	Dell et al. 2022	CA	Regional Psychiatric Centre	39 months	POR	NS	4	NS	NS	NS	NS
7	Gillard et al. 2009	UK	Mental health services	N/A	Public involvement	2	3	-	NS	NS	Yes
8	Kip et al. 2019	NL	Forensic hospital	NS	Participatory development process	2	2	3 therapists 1 policy advisor	convenience sampling, policy advisor initiated	NS	NS
9	Livingston et al. 2013	CA	SH, NCRMD	~ 2 years	PAR	NS	8	pre-existing Patient Advisory Committee	NS	NS	Yes
10	Livingston et al. 2013	CA	SH NCRMD	19 months	PAR	NS	8 (6 by the end) Team PEER, NCRMD,	1 peer support worker, pre-existing Patient Advisory Committee	NS	NS	Yes
11	Long et al. 2012	UK	LS & MS wards within women’s hospital (PD)	NS	user-led participatory research	2 researchers	1	1 service user involvement support worker	NS	NS	NS
12	Tearle et al. 2010	UK	Community service (ID forensic needs)	8 sessions	PAR	NS	1	1	NS	NS	NS
13	Visser et al. 2021	UK	LS & MS	NS	NS	2	2	-	NS	NS	NS
14	Wharewera-Mika et al. 2020	NZ	L-MS	NS	PAR	2 Māori researchers	1 Māori consumer advisor & 4 cultural workers	5 Māori, 2 non- Māori	NS	NS	NS

* Abbreviations: Low Secure (LS), Medium Secure (MS), High Secure (HS), Participatory action research (PAR), Facilitated Collaborative Action Research (FCAR), Patient oriented research (POR)

**Table 3:** Overview of other types of publications included in the review (reports, reviews, secondary analyses, and reflections)

No	Author, Year	Type	Aim	Focus
**1**	Faulkner 2006	Report	to capture experiences and to enable others to learn the lessons about involving service users in forensic mental health research.	reflections on four research projects
2	Feldbrugge 1981	Report	to set in motion an action research programme and discusses problems attendant with such an approach.	how action research could be received and employed in van der Hoeven Kliniek
3	Godin & Davies 2010	book chapter	to explore challenges and ethical issues in involving service users in research in the area of forensic mental healthcare	exploring policies and relevant research studies
4	Godin et al. 2007	retrospective consideration	how a research study opened up space for communicative action about forensic mental health care services.	reflections on the project and communicative action
5	Haarmans et al. 2021	Reflection	describe and reflect upon the initial process of setting up a participatory action research (PAR) project within an assessment and treatment service	the early stages of developing the participatory action research project up to the point of commencing the research
6	Halsall 2010	Book chapter	describe and reflect on a user-led research project in a medium secure unit	the whole research process, including preparation, funding, ethics application and challenges throughout
7	Inglis & Swain 2012	Secondary analysis	to explore how men with a learning difficulty understand research, consent and ethics	data from a project utilising participatory research were reanalysed using critical discourse analysis
8	MacInnes et al. 2011	Reflection	looks at the factors perceived by professionals and service-users as important for developing collaborative research in forensic mental health settings.	reflections on a collaborative research project undertaken in three forensic mental health units
9	Spiers et al. 2007	Editorial	an overview of service user involvement in forensic mental health, policy and practice	current state of service user involvement in forensic mental health researcher and the UK National Forensic Mental Health Research and Development Programme
10	Staley, 2013 (Case Study 3)	Case study	to capture the lessons learned from people’s experiences	exploring service user’s views on their involvement in research
11	Völlm et al. 2017	Rapid review	how best to engage users of forensic mental health services in the research process, and to make appropriate recommendations.	23 documents included in the review

### Level of involvement

The level of involvement [32] between and within studies varied, covering all the spectrum of the tool, with patients acting as listeners^7^ (giving information), co-thinkers (giving opinion opinion), advisors (giving unsolicited advice), partners (working as equal partners) to decision makers (taking initiatives/final decisions). Most studies have involved patients as advisors (giving unsolicited advice), partners (working as equal partners), or decision makers (taking initiative/final decisions) in the main stages (and tasks involved) of the research process. This was particularly true for the design of data collection strategy (e.g. tools, questions), recruitment and data collection. In data analysis, most studies involved forensic patients as advisors or partners, two as co-thinkers and three as listeners, while there was no study involving patient as decision makers, at this particular stage. The table below (Table 3) shows the level of involvement of each study at its various stages (e.g. in the study by Banongo et al. [48] PWLE were thought to be involved as partners in proposal/funding and ethics stages, but as decision makers at the dissemination/seminar presentations). The stages outlined in this table reflect the descriptions provided by the authors in their respective studies. It should be noted that as the tool was used retrospectively and relied on information provided on publications (Table 4).

**Table 4:** Patients’ involvement in research, Using the involvement matrix tool [32] to map the level of involvement

		Listener (is given information)	Co-thinker (is asked to give opinion)	Advisor (gives unsolicited advice)	Partner (works as an equal partner)	Decision-maker (takes initiative, (final) decision)
**Preparation**	Identify research idea	Abram et al., 2019 Alred, 2018 Cook & Inglis, 2012		Dell et al., 2022		
	Defining research questions		Alred, 2018	Dell et al., 2022		Banongo et al., 2006
	Proposal / Funding	Alred, 2018		Visser et al., 2021	Banongo et al., 2006	
	Ethics	Abram et al., 2019	Alred, 2018		Banongo et al., 2006	
Execution	Design materials (no instruments/interview guides)			Cook & Inglis, 2012	Cook & Inglis, 2008 Kip et al., 2019	
	Choosing / Developing Instruments and Questions			Dell et al., 2022 Visser et al., 2021	Dell et al., 2022 Gillard et al., 2009 Livingston, Nijdam-Jones & PEER, 2013	Abram et al., 2019 Long et al., 2012
	Recruitment			Visser et al., 2012		Long et al., 2012
	Data collection		Cook & Inglis, 2008 Tearle et al., 2010 (I)*	Dell et al., 2022 Tearle et al., 2010 (II)* Wharewera-Mika et al., 2020	Abram et al., 2019 Alred, 2018 Gillard et al., 2009 Tearle et al., 2010 (III)*	Banongo et al., 2006 Cook & Inglis, 2012 Livingston, Nijdam-Jones & PEER, 2013 Long et al., 2012
	Data analysis	Gillard et al., 2009 (I)* Visser et al., 2012 Wharewera-Mika et al., 2020	Abram et al., 2019 (I)* Dell et al., 2022	Abram et al., 2019 (II)* Cook & Inglis, 2012 (I)* Cook & Inglis, 2008 (I)* Gillard et al., 2009 (II)* Livingston, Nijdam-Jones & PEER, 2013 (I)*	Alred, 2018 Banongo et al., 2006 Cook & Inglis, 2012 (II)* Cook & Inglis, 2008 (II)* Gillard et al., 2009 (II)* Livingston, Nijdam-Jones & PEER, 2013 (II)* Long et al., 2012	
	Interpretation & validation of findings				Alred, 2018	
	Ongoing research design (evaluation and adaptation)		Cook & Inglis, 2012	Alred, 2018 Dell et al., 2022 (I)*	Cook & Inglis, 2008 Dell et al., 2022 (II)*	
Implementation	Report writing	Alred, 2018			Banongo et al., 2006	
	Publication	Tearle et al., 2010		Alred, 2018	Abram et al., 2019 Cook & Inglis, 2008 Livingston, Nijdam-Jones & PEER, 2013 Long et al., 2012	
	Other written material (brochures etc.)					Cook & Inglis, 2008 Livingston, Nijdam-Jones & PEER, 2013
	External presentation	Wharewera-Mika et al., 2020		Banongo et al., 2006	Alred, 2018 Cook & Inglis, 2008 Dell et al., 2022	
Other	Project coordination				Kip et al., 2019	
	Strategy development for further patient engagement				Dell et al., 2022	
	Seminar presentation for nurses education					Banongo et al., 2006

* Some studies contain more than one tasks in each phase (e.g. data analysis) in which forensic mental health patients were involved to varying degrees (e.g. co-thinker in one task and advisor in another task during the same phase). These varying levels are indicated by Roman numerals

### Reflexive thematic analysis

**Fig. 2:**
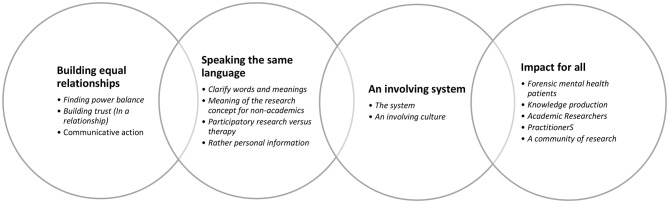
Themes & Subthemes emerging from the analysis

The analysis focused on identifying (i) helpful strategies with regard to participatory research in forensic mental health settings and (ii) the impact reported of using participatory research in such settings on PWLE, practitioners, settings, researchers and knowledge production. We identified the following themes (Fig. 2).

### Theme 1 – Building equal relationships

The difference between participatory research approaches and other more ‘traditional’ approaches of research were discussed in all papers included. With empowerment at its core, participatory research approaches aimed to actively involve people with lived experience in the knowledge production process. PAR provided a helpful opportunity for gaining a unique insight through directly accessing individuals’ lived experience.

Participatory research was described as not just a methodology to follow, it requires ‘*a longstanding interest and belief in the importance of service users having much more say…*’ [48] to ‘*enable all voices to be valued.’ *[51]*.* It is more like a *‘political commitment to working with*,* and addressing powerlessness*,* PAR [participatory action research] can*,* as KT has said*,* “amplify your voice.”* [9] *.* Although all studies and other publications portrayed this type of involvement of forensic mental health patients, it was not a straightforward task and required increased motivation and effort in comparison to other more ‘traditional’ forms of research.

#### Finding power balance

Within participatory research, the role of the academic researchers transformed from solely conducting or facilitating research to facilitating and encouraging open dialogue among the research group. For making participatory research work, ‘traditional’ roles within research contexts needed to change; and this was not an easy task. One helpful strategy was to clarify that there are no researchers and research subjects, but one research team. Similarly, monetary compensation was reported to be helpful in these power shifts as it further underlined the notion that all research group members receive compensation for their work.

As such, a power shift is commonly required, for making research a shared activity, where all research group members can (and are expected to) equally contribute to the research process.

*…the exchange of different perspectives (for mutual learning) should not be limited by prior power relationships in which the views of those perceived as having lower status or having less power are rendered inherently less valuable*,* less plausible*,* less useful and less well founded than the views of those with higher status. *[51]

The power shift is occurring within groups and forms (and is being formed by) interactions. In several cases, groups were asked to choose, as a group, the way that they will work together. This sometimes constituted the first task of the research group (e.g. [9, 52, 53]) while later they were being equally (more or less) involved in data collection and analysis. This equal involvement was thought to contribute to the building of a sense of ownership, which, as noted in several studies, is an important factor for active involvement and engagement [54–56]. This increased sense of ownership increased motivation and commitment.

#### Building trust (in a relationship)

Building a relationship and, most importantly trust, was a central point. Team members developed an actual relationship that was based on sharing and supporting each other, and this is what enabled and supported co-production. This relationship was similar to other relationships created in personal lives, where people share and celebrate important dates together [55]. Building trust seemed to be a prerequisite for effectively using participatory research. Moreover, one study, which had a focus on people coming from a different cultural background, discussed how meetings and the building of a relationship based on trust occurred in a culturally informed way, within ‘*Māori tikanga (culturally correct ways of doing things)’* [57]. It seems that adopting a genuine and culturally informed approach was especially important.

Building trust was described as a time-consuming and dynamic process, influenced by the setting within which it occurs. It was something that was developing throughout the course of the meetings and research projects [51]. Working together was not as enjoyable initially as it ended up being towards the mid or end of projects, where groups were well-established. Moreover, the forensic context and its restrictiveness were noted by some to act as a barrier in this trust building. This was particularly true for members of staff who were involved in the research group, and who had to comply with the settings’ strict policies (e.g. on sharing personal information), and not so much for the relationship-building between forensic mental health patients and academic researchers, who had some more freedom in making such decisions [9].

#### Communicative action

For participatory research to be fruitful, a ‘*conversation space’* needed to be created within the context of these ‘equal relationships’. A space where ‘*conversational partners’* can get involved in an ‘*open academic debate’* and move towards *‘communicative action’* [52, 55]. It was within communicative action, action oriented towards mutual understanding, that research occurred, and knowledge was co-created. This opportunity for equal ‘*dialectical discussions*’ [20] or ‘*free open debate’* [58] or ‘*open dialogue’* [59] led to ‘*in-depth understanding’* [20] and ‘*increased insight’* [55]. As scholars noted, ‘*This recursive approach*,* driven by the men’s need to get ‘‘to the heart of the matter’’’* [20] or ‘*collected critique’’* [51] created knowledge.

### Theme 2 – Speaking the same Language

It became apparent in some studies that not *‘everyone is always ‘speaking the same language’’* [52]. ‘*Seeing things from another person’s perspective* [54] was a demanding task. As such, it was helpful to put unique attention into language, and an effort to foster meaningful communication and promote the co-production of shared meaning was made. Academic language and the use of jargon acted as a barrier for participation. This was thought to be both due to unfamiliarity with scientific language and jargon (from forensic mental health patients’ side), but also due to power imbalances reflected in language.

*…the language commonly used in academic research can inhibit or destroy rapport when conducting research in prison by*,* for example*,* using wording that infers equality between groups*,* or using words like ‘informant’ that may have a different meaning within the prison community.* [27]

#### Clarify words and meanings

Words had different meanings to different members of the group, based on their personal histories, experience and knowledge. This was true not only for words related to research, but also other terms used to describe the everyday world and acts [48, 51]. As a result, involvement of forensic mental health patients in the analysis of data actually changed the interpretation of data collected through research and final outputs [54].

The importance of words and meaning was, in most cases, well-recognised, and lots of attention was put into terms used throughout the research process [12, 17, 56]. This attention resulted in changes in how things were communicated within groups and later with potential participants of the studies. Relevant research materials, such as posters, participant information sheets and consent forms were co-created, reflecting different styles and perspectives. Sometimes, other non-verbal forms of materials, such as pictures were used to facilitate conversation and aid understanding.

Although accessibility was still an issue, language decisions reflected this well-balanced collaboration, which required shifting away from ‘traditional’ academic language. One study stated that …co-researchers suggested replacing ‘*attain*’ with ‘*achieve*’ in the following section of the Participant Information Sheet…. For example, KT insisted on replacing the wording: ‘*Neither Agree or Disagree*’ with ‘*Unsure*’ [9]. Forensic mental health patients led this process of re-presenting research in a more accessible way for this particular setting and population.

Switching to a less academic/scientific language was also a challenging task for other reasons. One paper noted that the use of a language different than the one commonly used in National Healthcare System research plans documents, made the director of the unit more hesitant in supporting the research plan [59], which posed new issues.

#### Meaning of the research concept for non-academics

A good example to illustrate the importance of words and meaning might be research itself. Some authors stated that academics frequently discussed research as a ‘*rather abstract*’ [20] concept. This is important to consider, especially when people, who are less familiar with academic research, are involved in research activities. Some publications argued that forensic mental health patients had a limited understanding of research, while others highlighted how forensic mental health patients’ prior experience of being involved in more ‘traditional’ forms of research, as subjects, could act as an obstacle for understanding participatory research (and what it might entail).

Therefore, it was important and helpful for the research team to ensure that there was a level of understanding of the research, as used (and even maybe defined) in their studies. This was achieved through training, which in some cases was personalised, fitting into individuals’ needs, and works towards co-creating shared meanings that they could use and rely on. In one of the studies, research was parallelised by PWLE to a jigsaw puzzle, where researchers need to put all piece together [51]; a very different perspective on research.

#### Participatory research versus therapy

Participatory research was sometimes confused with therapy. Studies and other publications noted that it was important to help forensic mental health patients distinguish between therapy and research. This was important for ensuring that the nature and aims of research, but also expectations of commitment could be well understood. Forensic mental health patients sometimes perceived PAR as a ‘*form of treatment from the ‘softer end’’* [55], or could confuse *‘the difference between observation as a research method and observation as part of care and treatment’ *[51]*.* This last bit was particularly important considering the nature of forensic settings, where observation is part of the everyday life. Moreover, it was noted that it was important to make the distinction between formal therapy and research clear in this particular context, so to stress the voluntarily nature of getting involved in research. Partcipatory research, in addition to some therapeutic interventions, was an act not (forcibly) imposed to individuals [57]. However, making this distinction between therapy and research was more challenging, when involvement in research was part of forensic mental health patients’ care plan [58].

#### Rather personal information

The disclosure of personal information was an important aspect of forensic mental health patients’ involvement in research. The majority of papers discussed the meaning of disclosure for forensic mental health patients and how it could impact their understanding and willingness to be involved in research [48, 55], which has to be reflected upon. Individuals had not just disclosed but also shared ‘*rather personal’* information and experiences throughout the research process. The sharing of personal experiences seemed to shape the understanding of research, both prior and after involvement. In a sense, a more ‘personal’ involvement was required by forensic mental health patients, in comparison to academic researchers and staff.

### Theme 3 – An involving system

#### The system

Of great importance was how mental health services or ‘the system’ was understood and what it meant for forensic mental health patients, as well as how this might have regulated their interactions and role within this environment. Patients described that ‘the system’ [60] was all about power and authority. ‘The system’ did not only regulate power dynamics between service receivers and providers, but also among staff members. One of the studies discussed how senior staff and psychiatrists were unsupportive towards nurses [55].

Forensic settings are known for their highly restrictive and coercive nature. Doing participatory research within such settings seemed to be a rather complicated process, in which constant conflicts and dilemmas arose. The authors of one study included stated that *‘…negotiating the power dynamic in a culture that is essentially restrictive*,* and controlling is not straightforward.’* [28]. Forensic settings have well-established power dynamics. Shifting power dynamics within a group for the purposes of a research project might be difficult (and it needs flexibility and time). This was quite apparent in cases staff members were involved in the research process. How can a patient disagree with someone they rely on in their daily life? [51]. Nevertheless, it might be impossible to mitigate existing power imbalance(s) but just acknowledging and reflecting on those seemed to be an impactful and useful strategy [55].

A good illustration of how pervasive power dynamics work in forensic contexts was recruitment. Staff were, in several cases, involved in the recruitment process, by providing references or identifying suitable forensic mental health patients to take part in participatory research. This might have stressed the power imbalance, as professionals were the ones to make final decisions on forensic mental health patients’ involvement. In some cases, forensic mental health patients were excluded from taking part in research, as they thought to be *‘too unwell to approach’* by staff members [16]. Balancing ethics and safety was thought to be challenging, as in one study researchers wanted to avoid involvement, or control, of recruitment being on the hands of practitioners, but on the other hand, they needed to ensure safety for forensic mental health patients and others [48].

#### An involving culture

The dominant non-involving culture in forensic mental health services was something that came up in several studies and other publications. Due to their restrictive nature, forensic settings put a lot of restraints on participatory research, and barriers on forensic mental health patients’ effective and meaningful involvement in research. In fact, participatory research, as a process, comes in conflict with what was expected by forensic mental health patients in this non-involving, *‘modern rational strategy-orientated world*,* where open debate is stifled by concerns to* ‘*get the job done’’* [55].

Several studies discussed the practical difficulties and risks and ethical dilemmas that came up while doing participatory research within such settings. Some studies talked about the risks participatory research might pose to security and explained that forensic mental health settings might be unwilling to use such approaches requiring power sharing [50]. In a similar sense, ethical concerns were raised regarding whether forensic mental health patients have or should have such freedom. The authors of one study stated that *‘critics questioned whether it was right to allow people who had committed serious crimes to have a voice as they had curtailed the freedom of their victims’.* [58]

Additionally, confidentiality in forensic settings was key and is sometimes difficult to maintain [17]; as such, it constituted another practical challenge. Forensic settings are small communities with people often staying for long periods of time. Any breaches in confidentiality might have effects beyond the end of the research project on those communities, as well as individuals’ mental health (for example skills acquired by PWLE).

Moreover, the forensic context was thought to impact forensic mental health patients’ behaviour, making them less open to trust and sharing. This was true for both forensic mental health patients [50] and services [28]. Forensic mental health patients sometimes showed lack of interest to get involved to research, or increased reluctancy to join (for other reasons) and a lack of trust to researchers [50]. In several cases, more time and clear communication was needed for establishing trust [55].

It was interesting to see that in cases where a culture of more active involvement of forensic mental health patients in care existed, involvement in research was eased and more easily promoted (e.g. *The Regional Psychiatric Center’s approach can assist with fostering patients’ empowerment within the institution*,* which possibly made power-sharing and collaborative processes more achievable for our team.* [53]). As such, creating a culture of involvement seems to be key for easing participatory research in such settings.

### Theme 4 – Impact for all

All papers included discussed, in detail, the impact of involving forensic mental health patients in research. Studies discussed that involving forensic mental health patients impacted forensic mental health patients, researchers and practitioners, both on a personal and professional level. Additionally, the impact on knowledge production, as well as wider professional practice was discussed in some studies. This impact was suggested to be long-lasting, namely, going ‘*beyond (the projects’*) *own lifetime*’ [51].

Over time, forensic mental health patients managed to build strong research and presentation skills, through trainings and group discussions [51, 55]. In some cases, forensic mental health patients had the chance to undertake work and trainings accredited by educational institutions as part of those research projects (e.g. they received credit modules in courses) [51]. This might have acted as a *‘stepping stone to work’* [56] and future goals **‘***It also showed me a new path to travel with my writing.’* [48].

#### Forensic mental health patients

As a process, participatory research in forensic mental health hospitals was described as a positive experience, with individuals involved stating that this was something they enjoyed and would try again [20, 47, 51, 56]. Through their involvement in research, forensic mental health patients were perceived to have built self-esteem, confidence, a sense of value and worth, as well as particular skills. Those skills were not just research skills, but skills making them competent to set and achieve new goals in their lives. Research involvement was discussed as a ‘*self-transformation’* [9] journey for forensic mental health patients. All studies and other publications made references to empowerment, embracing skills and/or feelings of pride and accomplishment.

Spending time and working with others in those settings was assessed as equally important for forensic mental health patients. Patients stated that learning in a group was easier and more enjoyable as a process, while working in a group helped them build social skills [47, 56, 61]. Forensic mental health patients had the opportunity to reflect in a safe and supportive environment. They had the opportunity to think about their personal journeys and mental health issues, hear other peoples’ stories and reflect. They reported that this had an impact on their wellbeing and recovery journey. In this way research had a therapeutic impact on them. Involvement revealed a different *“possible future self”* [9] to some individuals, built hope and opened up new opportunities.

#### Knowledge production

Several studies discussed the impact of involving forensic mental health patients in research on the quality of the knowledge produced. Involvement was thought to increase trustworthiness, rigour and authenticity of knowledge produced, as it opened up space for more perspectives to be heard and considered [47, 62]. At the same time, participatory research was thought to make research more inclusive and knowledge production more accessible [25]. Involvement of forensic mental health patients supported recruitment of participants [60]. It widened its reach, as individuals, who might not have participated in research otherwise, did sometimes participate, due to forensic mental health patients’ proximity to others [55].

#### Academic researchers

In all of the studies and other publications, there was limited focus on the impact of participatory research on researchers, in comparison to the impact on forensic mental health patients, which was more widely discussed. However, participatory research was a positive endeavour for academic researchers too. ‘*…one researcher said it was ‘one of the most enjoyable pieces of research I’ve done’; another that the service users had exceeded expectations.’* [56]. Some studies talked about how participatory research shaped researchers’ identity and built on their understanding of the topic under investigation, whilst promoting more reflection, and as such a better insight (e.g. on the research process and researcher’s impact and stance) [52, 55].

#### Practitioners

Similar to academic researchers, there were fewer references to the impact of participatory research on practitioners and professional practice (e.g. [51, 56]). This might be explained by the lack of involvement of practitioners in many studies involved in this review. Studies that have involved practitioners talked about how participatory research enriched the role of practitioners and opened space for more meaningful and ‘positive’ communication between practitioners and forensic mental health patients [51]. Practitioners were allowed to see a different side of forensic mental health patients commonly ignored; forensic mental health patients were full of capabilities and potentials [56].

In terms of research skills, some practitioners felt that they did not build any new knowledge or skills through their involvement in participatory research [51]. However, the level of involvement seemed to have played an important role on their judgements, with people more actively involved in group work and discussions feeling that they have actually acquired new skills.

#### A community of research

There were discussions on how participatory research can contribute to the development of a community of research. Participatory research was thought capable of ‘building bridges’ between academic researchers, forensic mental health patients and services, by creating connections and alliances, throughout the course of research projects [56, 61, 62]. By creating this community of research, where different voices are being heard, we might be in a better position to ensure that services are better designed for particular groups of patients (e.g. minority groups). In this way, services might be better accepted and consequently increase their effectiveness.

## Discussion

Our review shows that, in line with previous scholarly work, building equal, genuine relationships, ensuring to speak the same language, and an involving system are fundamental in ensuring a noticeable perceived impact for all with respect to participatory research in forensic settings [63]. PWLE’s involvement cannot be seen as a strictly professional task, and participatory research works more as a philosophy rather than just a research approach [3]. PWLE are frequently asked to bring a ‘rather personal’ and private side of themselves into the projects. This is why genuine relationships and trust are especially important in forensic settings.

Regarding building equal relationships, monetary compensation of patient researchers seemed to be a helpful way to recognise their contribution in the work produced [22]. Payment, as well as the type of employment of patient researchers was not always described adequately (including payment) and/or employment seemed to act as a challenge in forensic contexts [28]. It was mainly the studies involving community members (out-patients and people with past experience of forensic mental health care) that elaborated on those aspects (e.g [47, 48]. Considering the nature of the context, and the practical restrictions it might imply, it is important to have a better overview of different helpful strategies with respect to employment and payment.

Language seemed to be a detrimental point for consideration, in line with what scholarly work in wider mental health suggests (e.g. [34]). Participatory research is commonly rooted within a social constructionist paradigm, and as such emphasises exploring subjective realities and intersubjectivities [64]. It seemed that continuous efforts were required to ensure that words and meanings were explored and co-created, when needed, to ease communication and avoid confusion. This is of relevance to this particular context, as was revealed in this review. Forensic mental health patients’ understanding of ‘research’ and ‘the system’ and its potential impact on their willingness or ability to make an informed decision on getting involved in research, might be some good examples to illustrate the importance of putting attention to language, words and meanings (e.g. [20, 28, 48, 51, 60]).

Forensic mental health settings as institutions were described as non-involving in nature, due to the increased restrictiveness. Institutional (and potentially systemic) power imbalances (e, g. [47, 52]) might impact the way that participatory research can be used in such settings, while they might also raise ethical questions and dilemmas. Forensic settings serve a dual purpose, they are rehabilitative and punitive settings. As such, from a punitive perspective some studies have questioned whether sharing power, and allowing more freedom to PWLE, a cornerstone for participatory research, is possible or even appropriate (e.g. [58]). Several authors talked about the lack of trust in such settings and its potential impact on studies [28, 50]. This is particularly relevant considering the frequent involvement of forensic mental health staff in recruitment, which is another topic that came up (e.g. [48]). Although possibly appropriate for security purposes, it shows an inherent power imbalance in cases where forensic mental health staff are the ones taking final decisions on whether people can (or are able to) participate in research.

In line with previous research in using participatory research with marginalised communities (e.g. [13–19]), all publications identified some perceived impacts for PWLE, researchers and the academic community, knowledge production, practitioners, and services. It was interesting to see how skills built through participatory research might be transferable to care, and how this opportunity for collaboration might work towards building a ‘research community’ mindset (e.g [61, 62].

All papers included reported an impact for all involved when working with PWLE in research. These impacts were reported to stand above the research aims and participatory research was often perceived as a form of therapy itself by PWLE (e.g. [, ]). This is of particular value, considering that commonly the aim of research is to increase the effectiveness of forensic mental health services. Additionally, in studies where practitioners/services’ staff members were involved, the involvement of PWLE in research allowed them to see PWLE as individuals with capabilities and skills, something often neglected in such settings. As such, it could be suggested that participatory research approaches could act towards reshaping the restrictiveness of forensic psychiatric settings, by, as suggested by the authors, building on the ‘*reflexivity by staff and policy-makers between the level of restrictiveness and security needed to safely provide care in a therapeutic milieu and enabling the maximum amount of resident autonomy’* [30].

Although perceived impacts were also identified for practitioners, researchers, the services themselves, as well as knowledge production (e.g. participatory research approaches allowed practitioners and researchers to get a unique insight into the topic under investigation), most studies and other publications focused on the impacts as perceived by patients. This is important, as it might be particularly useful for academic institutions, and funders to have a better overview of wider impacts, so they can make better informed decisions (e.g. [, ]). Additionally, reporting perceived impacts for all sides might act as evidenceof acknowledging and mitigating power imbalances.

Although the majority of studies reflected efforts to involve forensic patients in all different stages of the research process, the level of involvement varied. This finding was in line with Hoekstra et al. [12] who suggested that different levels of involvement might be required in different contexts and different research stages. It was thought that in some cases different levels of involvement were allowed (instead of chosen) in different stages, and that was mainly attributed to the nature of forensic settings [30]. However, we would like to highlight that our mapping was done retrospectively from an outsider’s viewpoint, and we had to rely on the information available in the publications. As such, it constitutes an indication rather than a mere fact where involvement might have ranged, and a good starting point for further discussion.

Finally, reporting of participatory research varied highly across the papers included. Although reporting guidelines for participatory research have been developed, (e.g [65]. there was a lack of uniformity in reporting. In line with what previous research suggested [66], often the focus was set on particular aspects of the study, while sometimes other elements were underreported (e.g. most studies have provided limited information on payment and training). Even though, to some extent, this could be anticipated, due to the complexity of participatory research projects and the different elements involved, this lack of uniformity might render participatory research less transparent. This is also relevant to critical appraisal of the quality of studies. As such, we suggest that a greater focus on reporting is important in future research, while the design and use of more appropriate tools (that would be able to capture the complexity of participatory research) for assessing the quality of studies would also be beneficial.

### Limitations

As several different approaches of participatory research have been developed, and different terms are being used in the literature to refer to participatory research, this systematic review might not have captured all participatory research papers in forensic settings. When designing the search strategy, all efforts were made to ensure that different approaches were captured (e.g. use of different terms, see search strategy). To avoid confusion, this review excluded other methodologies that require the involvement of patients to certain stages of research, such as photovoice and Delphi studies. However, as those studies could be considered as part of a wider participatory research family, involving those types of studies could have enriched the review and its outputs.

In addition, forensic mental health care is practised in different contexts, for example in forensic hospitals and special units within prison settings. Although we developed the research strategy in a way that studies in all such settings would be captured, during the reviewing stage it was realised that due to the great variation in the terms used to describe those units, this could be a bigger challenge than originally anticipated. As such, this review might have missed some studies in units within prisons.

Furthermore, considering that most studies employed convenience or purposive sampling (e.g. [51, 67]), and involved clinical members of staff in their selection (either for suggesting potential participants or for providing clinical references), we believe that our current view might be ‘restrained’. Employing a wide range of sampling techniques might help to broaden our understanding on this topic. For example, ensuring that PWLE from diverse backgrounds and different needs are involved in research, and utilising different sampling approaches might be some strategies to explore. This, however, would need to be done in a way that would not jeopardise services or pose risks to patients and their wellbeing (i.e. considering the impact of involving people with different needs on teams).

The sampling approach usually employed, might also build on power imbalance, as the process places practitioners and/or academic researchers in a powerful position of choosing PWLE. This is something that might come in conflict with participatory research, and as such undermine it. However, this is difficult to avoid. Future research should explore ways to overcome this by employing different strategies, for example allowing PWLE to explain how they can deal with/overcome certain characteristics that are perceived to act as barriers for their involvement to research.

Most studies lacked in diversity; this was relevant for both PWLE and academic researchers. Most studies in the sample of this review involved white males as patient researchers, and white males and females as academic researchers. However, information on the profiles of researchers was often limited, with only a small part of the studies providing a description of the academic researchers’ profiles (e.g [9]). Furthermore, all studies were conducted in the Global North, with most in the UK [8, 17, 20, 47, 48, 51, 52, 54]), Canada [49, 50, 53] one in the Netherlands [67] and one in New Zealand [57]. As such, we might still have a limited understanding of how participatory research can be applied in different socio-cultural and legal contexts.

While some studies described the training provided to members of the research team (e.g. [51, 62]), others included limited or no information. Due to the complexity of participatory research projects, the detailed reporting of all different aspects and stages might be challenging, however, information on training, both content and structure, might be important for future research and transparency. Inadequate training and preparation might diminish the benefits of using participatory research.

## Conclusions

It became apparent from this review that forensic settings, which are restrictive in nature (28 30), might, if not appropriate considerations are made and measurements taken, limit, diminish or even disregard participatory research’s ultimate goals, empowerment, and social change. This is in line with what other scholars have suggested [34]. As such, careful planning of involvement, needs and challenges that might arise in such contexts, prior to the start of research, is crucial. This review identified helpful strategies with regard to participatory research in forensic mental health settings and the impact reported of using participatory research in such settings on PWLE, practitioners, settings, researchers and knowledge production. We also listed some common challenges, good practices, and ‘*active ingredients’* in the involvement of forensic mental health patients in research.

### Implications for future research

This review included mainly qualitative studies and reports. Involving forensic mental health patients in research, as a process, would be the same regardless of the nature of the research itself. However, actively involving forensic mental health patients in quantitative research might reveal different issues, and challenges (e.g. we would assume that more training could be required). Future research might also use quantitative methods to demonstrate the impact of involving service users, e.g. on the quality of research, service users, etc.

Considering the range of the level of involvement of forensic patients throughout the different stages of research (as identified retrospectively in this review), and in line with what scholars have previously suggested [31], we believe that it is important for future research to consider employing tools (or ways) to map involvement. This would allow the research team (academic researchers, patients researchers and professionals) to get a more accurate overview of patients’ involvement, while also adding to transparency, and helping patient researchers to make informed choices.

In addition, diversity might be a key issue for future research. More efforts should be made to ensure that people from diverse backgrounds are involved in participatory research, and that participatory research approaches are applied in different socio-cultural and legal contexts. This might provide us with a broader picture of the capabilities of participatory research, the potential impacts, challenges, and barriers to full implementation in forensic settings.

## Supplementary Information

Below is the link to the electronic supplementary material.

Supplementary Material 1

Supplementary Material 2

## Data Availability

No datasets were generated or analysed during the current study.

## Footnotes

1 We used the term ‘forensic mental health patients’ for our review as people in the studies included were either in or out-patients. Even though we acknowledge that the term ‘patients’ might misrepresent people [29], we felt that it might ease communication in this manuscript. In the last parts of this manuscript (e.g. discussion, identification of active ingredients, Appendix B) we have decided to switch to PWLE (People With Lived Experience), as we thought that this term would better capture people who have past experience of forensic mental health care and could be involved in research in this field. Again, the use of PWLE might also pose risks [2].

2 Different tools for measuring the level of involvement have been developed and applied in practice, focusing on different aspects of involvement (e.g. Arnstein’s participation ladder focusing on power) (e.g. [31–33]).

3 We have used the term *tokenism* based on Arnstein’s participation ladder [31], where the lowest section of the ladder is non-participation, followed by tokenism, and then citizen control. *Tokenism* constitutes a non-genuine and non-meaningful involvement of PWLE in research.

4 Individuals (and or family members) with past experience of staying in secure hospitals or psychiatric units within the prison system and receiving forensic psychiatric care were also included.

5 In both screening stages, a random 20% sample of records was independently screened by a second reviewer to assess consistency [41]. This approach was informed by the high level of agreement observed during the initial screening phase. Inter-rater reliability was not formally quantified; however, disagreements were infrequent, minor in nature, and resolved through discussion. It was predefined that the proportion of double-screened records would be increased, or a third reviewer involved, if substantial disagreement had emerged at any stage of the review. Given the clearly defined inclusion and exclusion criteria, the limited number of eligible studies, and the consistency observed during the screening process, partial dual screening was considered sufficient to ensure the accuracy and robustness of study selection.

6 The term *‘active ingredients’* has been adopted by Abram et al. [47]. In fact, the idea to include this list was inspired by this work and its relevant recommendations for future research. This review aimed to guide next stages of this research project (Part-Beirat, 37) aiming at utilising participatory research approaches with forensic mental health patients, and as such, a list with ‘do’s and don’ts’ on practical matters thought to be of particular benefit.

7 The term listener, similar to co-thinker, and decision maker, derives from the involvement matrix [32] and reflects the different levels of involvement in research, it should not be confused with the way this term is being used sometimes in custodial settings referring to peer support roles.

## Abbreviations

PR: Participatory Research
PAR: Participatory Action Research
CBPR: Community-Based Participatory Research
PPI: Patient and Public Involvement
PPIE: Patient and Public Involvement and Engagement
FCAR: Facilitated Collaborative Action Research
POR: Patient oriented research
PWLE: People With Lived Experience
LS: Low Secure
MS: Medium Secure
HS: High Secure
NCRMD: Not Criminally Responsible on account of Mental Disorder
NGRI: Not Guilty by Reason of Insanity
PRISMA: Preferred Reporting Items for Systematic Reviews and Meta-Analyses
MMAT: Mixed Methods Appraisal Tool
