# Supplementary material for: A systematic review of participatory research involving forensic mental health patients
Source: BMC Psychiatry. 2026 Jan 23;26:85. doi: 10.1186/s12888-026-07813-8 (PMC12849659; doi:10.1186/s12888-026-07813-8)
Supplement: Supplementary file 1 — Supplementary Material 1 [file 12888_2026_7813_MOESM1_ESM.docx]

# Appendices

## Appendix A

MMAT

|  |  |  |  |  |  |  |  |  |  |  |
| --- | --- | --- | --- | --- | --- | --- | --- | --- | --- | --- |
| **No** | **Author/Title** | **Screening Questions** | | **Study Design** | **Study design specific Qs** | | | | | **Overall Score** |
|  |  | **S1** | **S2** |  | **1.1** | **1.2** | **1.3** | **1.4** | **1.5** |  |
| **1** | **Livingston et al.** | Yes | Yes | Mixed Methods | Yes | Yes | Yes | Yes | Yes | 5***** |

CASP

|  |  | **Sections** | | | | | | | | | |  |  |
| --- | --- | --- | --- | --- | --- | --- | --- | --- | --- | --- | --- | --- | --- |
| **No** | **Author** | **A** | | | | | | **B** | | | **C** | **Appraisal Summary** | |
|  |  | **Q1** | **Q2** | **Q3** | **Q4** | **Q5** | **Q6** | **Q7** | **Q8** | **Q9** | **Q10** | **High - moderate** | **Moderate- low** |
| 1 | Abram et al. | Yes | Yes | Yes | Yes | Yes | Yes | Yes | Yes | Yes | Yes | x |  |
| 2 | Alred | Yes | Yes | Yes | Yes | Yes | Yes | Yes | Yes | Yes | Yes | x |  |
| 3 | Banongo et al. | Yes | Yes | Yes | Yes | Yes | Yes | Yes | Yes | Yes | Yes | x |  |
| 4 | Cook & Inglis | Yes | Yes | Yes | Can't tell | Yes | Can't tell | Yes | Yes | Yes | Yes | x |  |
| 5 | Cook & Inglis | Yes | Yes | Yes | Yes | Yes | Can't tell | Yes | Yes | Yes | Yes | x |  |
| 6 | Dell et al. | Yes | Yes | Yes | Can't tell | Yes | Can't tell | Yes | Yes | Yes | Yes | x |  |
| 7 | Gillard et al. | Yes | Yes | Yes | Can't tell | Yes | Yes | Yes | Yes | Yes | Yes | x |  |
| 8 | Kip et al. | Yes | Yes | Yes | Can't tell | Yes | Can't tell | Can't tell | Yes | Yes | Yes | x |  |
| 9 | Livingston et al | Yes | Yes | Yes | Yes | Yes | Can't tell | Yes | Yes | Yes | Yes | x |  |
| 10 | Long et al. | Yes | Yes | Yes | Can't tell | Yes | Can't tell | Yes | Yes | Yes | Yes | x |  |
| 11 | Tearle et al. | Yes | Yes | Yes | Can't tell | Can't tell | Can't tell | Yes | Can't tell | Yes | Yes |  | x |
| 12 | Visser et al. | Yes | Yes | Yes | Can't tell | Yes | Can't tell | Yes | Yes | Yes | Yes | x |  |
| 13 | Wharewera-Mika et al. | Yes | Yes | Yes | Can't tell | Yes | Can't tell | Yes | Yes | Yes | Yes | x |  |

## Appendix B

| **Active ingredients and things to consider** | |
| --- | --- |
| **Overall** | 1. It takes effort. time and money. 2. Some useful skills: interest, enthusiasm, passion and compassion 3. Ensuring understanding and informed choices is vital throughout the whole process. 4. It needs flexibility and openness. 5. Transparency is important. 6. Involvement is a dynamic process -group dynamics and roles will be evolving. 7. Everyone has their own pace. 8. The involvement of PWLE in all stages is beneficial. 9. The researcher role is different than “traditional research” -acting more like a facilitator and an expert guide to research methods. 10. Setting clear goals -rewards and achievements, 11. Assessments prior to the start of the project are useful - assessing risks prior to start of the study, assessing teams’ needs and readiness for engagement (e.g., attitudes, resources, flexibility). 12. Ownership is important – and leads to higher commitment. 13. Make sure everyone is on the same page- co-designed protocols, ground rules, a dictionary of terms. 14. Being creative -using pictures, DVDs, flip charts and other alternative means for initiating discussion, training, research. 15. It might be beneficial to involve services -services might need more incentives to get involved. 16. Engaging and educating front line staff -helpful but sometimes challenging. 17. End of project evaluation – even though it might be difficult to put things into numbers. 18. Coming to an end – be prepared. |
| **Funding** | 1. More resources and time are usually required in comparison to “traditional research“ that funders might be more familair with. 2. Flexibility is required -as research plan and design require the involvement of PWLEit might be difficult to have a detailed research plan -to be submitted for funding and ethics. 3. Considering the potential benefits to the wider society and services. |
| **Ethical considerations** | 1. Focus on voluntary participation -a potentially challenging task with coercive settings. 2. Keep participants and PWLEs well informed at all stages (e.g. provide interview questions in advance, provide transcripts of interviews). 3. Confidentiality is key and might need extra considerations to be made-everyone knows everyone within forensic hospitals -maintaining confidentiality and anonymity even after the end of the study (e.g. long-term patients). 4. Privacy and confidentiality from staff members –difficult. 5. Doing research within a coercive setting -how it might come in conflict with this approach. 6. The impact of research to PWLEs as researchers needs to be also considered -in addition to the impact on academic researchers. 7. When participation was part of PWLE’s care plan ethical considerations arose -sharing, confidentiality, power imbalance-, while also could blur the distinction between research and therapy |
| **Employment** | 1. A detailed job description helps – to clarify expectations, duties. 2. Criminal history might act as a barrier for any time of employment. 3. Documentation required for employment might be hard to get (e.g. birth certificate). 4. References might act as a barrier -people’s reluctancy to ask references. 5. Payment is important (“money shows respect“), but might be challeging in forensic settings 6. Payment -how much and in what basis -following guidance (e.g. A Fair Day’s Pay (Scott 2003). 7. Payments not affecting benefits -or any kind of state support. 8. Payment – think about access to bank accounts, bureaucracy and delays. |
| **Informed consent** | 1. Consider the education level and profile of people you seek to involve (PWLE, participants etc). 2. Using different formats to present information 3. People need to understand the potential impact of research for providing informed consent. 4. Staff members and other professionals might be asked to give their ‘clinical consent’ for PWLE to participate in participatory research. |
| **Forming the research group** | 1. Early involvement is important and useful -identifying research priorities, recruitment. 2. Think about recruitment strategy -feasibility, risks and inclusivity. 3. Consider involving members of staff and/or key workers in identifying suitable candidates. 4. Important to get the right mix.    1. A balanced number of PWLE, academics and stakeholders.    2. Try to involve people from different backgrounds (e.g. ethnic, religion, gender, age) 5. A good balance between the number of members and amount of work to be done. 6. Identifying a staff liaison person -to liaise communication between the research team and the services. 7. Check how PWLE will be referred as – check with people involved. 8. Come up with a group name -building collective identity. 9. PWLE who knew each other before – easier to build rapport and connection . 10. Even though it might be difficult to ensure stability, efforts should be made for it – e.g. release to community, fluctuations in mental health. 11. Having new members later in the group will change group dynamics and the group members will need time to build rapport. 12. Providing adequate orientation for new members of the research team. |
| **Mental health** | 1. Mental health might sometimes act as a barrier for full participation. 2. You have to acknowledge the mental health difficulties that people may have and realise they may not always be totally motivated. 3. For some PWLEs joining the group and the discussions later on might be stressful and trigger mental health issues. 4. Considering how specific periods might impact individuals’ mental health (e.g. Christmas and alcohol issues). 5. Continuous monitoring of mental health. 6. Be ready to signpost to services for support, if and when required. |
| **Finding the right words** | 1. Language matters – might act as a barrier or promote involvement. 2. Co-creation is important (e.g. how things should be asked from an academic research perspective vs insider perspective). 3. Flexibility -allow people to express in their own ways (e.g. keeping diaries in different formats suitable to individual’s needs and personal style). |
| **Meetings** | 1. Regular meetings & regular breaks. 2. Length -consider content, purpose of meeting and individuals’ needs. 3. Refreshments and snacks are important – creating a relaxed and safe environment. 4. Expect disruptions and delays due to the nature of the setting. 5. Guards and other staff members might be required to be involved – e.g. accompany experts by experiences - be aware of conflicting roles. It might be good if prison officers who accompany PWLEs had expressed interest themselves in the group. 6. The first meetings -building rapport. 7. Shifting power takes time and effort. 8. Discussion in cycles (planning, action, reflection, evaluation). 9. Meetings require flexibility and will be dynamic (roles, power). 10. Meetings can be fun, but also challenging and boring -breaking in small chunks. 11. Even though there needs to be a focus on particular topic, cautious not to limit PWLEs’ ability to share their personal experiences. 12. When staff members are involved might ease or complicate communication and power dynamics -act as gatekeepers. 13. Not everyone will feel the same in the meetings (e.g. some might be at ease and others having issues) – ensure to check how everyone feels. 14. Raising rather than suggesting answers. 15. Consider splitting the group for allowing quiet members to be heard. 16. Ready to deal with frustration – and mediate. 17. Using regular resumés -what happened in the previous meeting. 18. Meetings might be the only opportunity to communication -communication outside meetings with PWLE is sometimes challenging and limited in forensic contexts. |
| **Where and when to meet** | 1. Aim for comfortable physical surroundings. 2. A neutral place (e.g. university setting) might help with building rapport. 3. Finding a suitable time -not conflicting with people’s others activities. 4. Commuting might be more complex than thought for some people (e.g. economic reasons, mental health, safety etc) -consider when planning meetings or interviews. 5. Allow flexibility, if possible. |
| **Rapport** | 1. Rapport needs time especially in forensic settings -involve people early. 2. Personal experience disclosure by academics and stakeholders promoting trust. 3. Allow (and create) space for sharing similar experience and future goals. 4. There needs to be room for both negative and positive emotions. 5. If other people are attending the meetings, involve them somehow -so they can build rapport with the group. |
| **Disclosure** | PWLE   1. Well established rapport increase individuals‘ willingness to disclose personal experiences. 2. Personal narratives might be used to initiate group discussion. 3. Might trigger emotions and thoughts (on a both individual and group level).   Academics and professionals’ disclosure   1. Need to be cautious with personal disclosure -can be helpful but also problematic (e.g. focusing on achievements). 2. It might be useful to talk about problem-solving strategies and skills. |
| **Training** | 1. Making learning a fun experience. 2. Flexibility -expect a variation in educational level, learning styles and needs. 3. Ensure training for all (experts by experience, academic researchers, stakeholders).    1. An individual assessment for training might be useful, as not all individuals will be on the same level and/or have same needs.    2. Academic researchers and stakeholders might need information on the forensic context (e.g. risks, security issues, population). 4. Enabling understanding instead of delivering info. 5. Be cautious with the use of technology. 1.not everyone will be tech-savvy enough), 2. Lack of equipment and resources -prior planning and finding ways to work with what you have 6. Making things visual might help (e.g. research cycle diagram). 7. Collaborative practice -helping other people to learn promotes own learning. 8. Group discussion, role playing, activities in small groups. 9. Practice helps and clarifies expectations (e.g. conducting practice interviews). |
| **Continuous support & reflexive practice** | 1. Continuous support is important -PWLEs might have no prior research experience. 2. Support offered in both group and one-to-one basis. 3. Practical support (e.g. reaching meetings place, liaison with staff members). 4. Checking points are important -for identifying any issues, reflection. 5. Make use of reflexive practices. Keeping track and sharing what is happening -such as check-out noted, debriefs, personal diaries, reflexive journals, field notes. |
| **Data collection and analysis** | 1. Discussing research design -advantages and disadvantages of using different methodological approaches. 2. Ensure a good balance between feasibility and complexity. 3. Involving PWLEs in recruitment & data collection -increasing quality & reach. 4. Allow all members to take initiatives. 5. Working on data analysis as a group. 6. Working on transcripts “the old way” – highlighters and pens -discuss comments in meetings. 7. Data analysis might be overwhelming especially at the beginning – allow adequate time and try to focus at one thing ( e.g.theme) at a time. 8. Group evaluation -triangulation of data. |
| **Dissemination** | 1. The contribution of everyone should be acknowledged. 2. Involve PWLEs in dissemination as early as possible. 3. Being involved in conferences and oral presentations -might be rewarding, informative & build ownership. 4. Make sure PWLEs are ready and willing to get involved in dissemination. 5. The order of the names in outputs should be considered and discussed (potentially different to “traditional research”, alphabetic order). 6. Dealing with reviewers’ negative comments might be a challenge - work on comments and revisions constructively as a group. 7. Findings -dissemination in accessible ways -everyone able to have a full copy of report. |
